# Robust Infrared–Visible Fusion Imaging with Decoupled Semantic Segmentation Network

**DOI:** 10.3390/s25092646

**Published:** 2025-04-22

**Authors:** Xuhui Zhang, Yunpeng Yin, Zhuowei Wang, Heng Wu, Lianglun Cheng, Aimin Yang, Genping Zhao

**Affiliations:** 1 Guangdong Provincial Key Laboratory of Cyber-Physical System, Guangdong University of Technology, Guangzhou 510006, China; xhzhang2022@mail2.gdut.edu.cn (X.Z.); yypeng@mail2.gdut.edu.cn (Y.Y.); llcheng@gdut.edu.cn (L.C.); amyang@gdut.edu.cn (A.Y.); genping.zhao@gdut.edu.cn (G.Z.); 2 School of Computer, Guangdong University of Technology, Guangzhou 510006, China; 3 School of Automation, Guangdong University of Technology, Guangzhou 510006, China; 4 Lingnan Normal University, Zhanjiang 524000, China

**Keywords:** infrared and visible sensors, image fusion, deep learning, image processing

## Abstract

The fusion of infrared and visible images provides complementary information from both modalities and has been widely used in surveillance, military, and other fields. However, most of the available fusion methods have only been evaluated with subjective metrics of visual quality of the fused images, which are often independent of the following relevant high-level visual tasks. Moreover, as a useful technique especially used in low-light scenarios, the effect of low-light conditions on the fusion result has not been well-addressed yet. To address these challenges, a decoupled and semantic segmentation-driven infrared and visible image fusion network is proposed in this paper, which connects both image fusion and the downstream task to drive the network to be optimized. Firstly, a cross-modality transformer fusion module is designed to learn rich hierarchical feature representations. Secondly, a semantic-driven fusion module is developed to enhance the key features of prominent targets. Thirdly, a weighted fusion strategy is adopted to automatically adjust the fusion weights of different modality features. This effectively merges the thermal characteristics from infrared images and detailed information from visible images. Additionally, we design a refined loss function that employs the decoupling network to constrain the pixel distributions in the fused images and produce more-natural fusion images. To evaluate the robustness and generalization of the proposed method in practical challenge applications, a Maritime Infrared and Visible (MIV) dataset is created and verified for maritime environmental perception, which will be made available soon. The experimental results from both widely used public datasets and the practically collected MIV dataset highlight the notable strengths of the proposed method with the best-ranking quality metrics among its counterparts. Of more importance, the fusion image achieved with the proposed method has over 96% target detection accuracy and a dominant high mAP@[50:95] value that far surpasses all the competitors.

## 1. Introduction

The images of visible sensors contain rich texture details, but they face limitations in low-light or night-time conditions. In contrast, infrared sensors capture the thermal radiation of a target for imaging, providing valuable information in dark environments. However, infrared images are often disturbed by noise. To fully leverage the complementary advantages of these two modalities, many infrared and visible image fusion (IVIF) methods have been developed to obtain comprehensive information about a target scene. The fusion of infrared and visible images (IVIs) has been widely applied in advanced visual tasks, such as target detection [1,2], tracking [3,4], and segmentation [5,6].

Over the past few decades, numerous IVIF methods have been designed, from traditional methods [7,8,9,10,11,12,13] gradually updating to deep-learning-based methods [14,15,16,17,18,19,20,21]. Traditional IVIF techniques can handle the fusion problem for scenes with idea imaging conditions, such as multi-scale transformation associated methods [7,8], sparse-representation-based approaches [9,10], methods depend on subspace clustering [11], saliency [12], and hybrid methods [13]. Nevertheless, the traditional methods mentioned above rely on manually set fusion rules and are unable to adapt to complex scenarios. Although one method [22] integrates multi-scale decomposition, sparse representation, and guided filtering techniques to improve the quality of fused images, it tends to have high computational complexity. In recent years, the advancement of deep learning has spurred the development of neural-network-based IVIF methods, which provide better results fonr IVIF tasks.

Deep-learning-based fusion approaches can be generally classified into three categories: auto-encoder (AE)-associated networks [14,15,16,17], convolutional neural networks (CNNs) [18,19], and generative adversarial networks (GAN) [20,21]. Specifically, AE-based IVIF methods reconstruct fused images through an encoder, feature fusion, and a decoder [14,15]. In fact, these methods also require manually designed fusion rules (concatenation, element-wise addition, etc.) [16,17] and cannot flexibly adjust the fusion content according to the input data characteristics. Therefore, the strategy for automatically adjusting fusion weights is of great importance for producing more robust fusion results. Additionally, some methods try to extract detailed information from the two modalities through different feature learning modules. For example, Qi et al. [18] guided the fusion process by learning sharp and blurry regions in the images. LRRNet constructs a learnable model by utilizing the low-rank and sparse coefficients [19]. However, these methods focus on enhancing the visual quality of the fused image, neglecting the guidance of semantic tasks. The acquired fused images also have difficulty in accommodating sophisticated visual challenges. Although some methods have tried to combine high-level visual tasks to guide fusion metrics, such as SeAFusion [23] and DetFusion [24], they do not account for the potential different feature representations between the fused image and the original images. This leads to artifacts in the fused image. So, some GAN-based methods, such as TGFuse [25] and AT-GAN [26], enhance the fusion effect by identifying specific features and removing redundant features. The discrimination between the fused image and the original images leads to the fused image containing more detailed information. However, the training of GAN is often unstable and requires careful loss adjustment to converge. In addition, the currently available public datasets for IVIF primarily contain terrestrial environments such as streets and roads, lacking diverse samples for special scenarios. As a result, the robustness and generalization of the related fusion methods are limited in practical applications.

To address the issues mentioned above, we propose an IVIF method based on a decoupled and semantic segmentation-driven network (DSSFusion). Firstly, we designed a cross-modality transformer fusion module (CMTFM) based on transformer cross-attention for learning rich hierarchical feature representations at multiple scales. Secondly, we explored a semantic-driven fusion module (SDFM), which incorporates semantic segmentation information to better preserve and enhance critical features of target objects. Thirdly, according to the characteristics of infrared and visible features, we adopted a weighted fusion approach that automatically adjusts fusion weights. This helps integrate the thermal features from infrared images with the structure and detail information from visible images. Subsequently, we designed a refined loss. In addition to the common gradient and pixel losses, we proposed an auxiliary loss via the decoupling network to constrain the pixel distribution between the fused image and the original images, resulting in more natural fused images. Based on the above design, our fused images maintain the texture of visible images while highlighting prominent targets regardless of lighting conditions. Finally, to validate the performance of the proposed method in complex scenes, we developed an aligned MIV dataset. The MIV dataset not only fills the gap in maritime scene datasets but also provides a new benchmark for evaluating fusion methods.

The main contributions of this paper are summarized as follows:

- We constructed an IVIF method based on a decoupled and semantic segmentation-driven network, which can automatically adjust the fusion weights and integrate meaningful information from the IVIs.
- We developed two fusion modules, namely, CMTFM and SDFM. These modules integrate information from different modalities by combining the transformer cross-attention mechanism with semantic segmentation, thus embedding semantic information into the fusion process.
- A refined loss function was designed to improve the quality of the fused images by importing auxiliary losses through the decoupling network to guide the training of the fusion network.
- We constructed a new benchmark dataset for evaluating infrared and visible image fusion in a special scenario called the Maritime Infrared and Visible (MIV) dataset. The benchmark dataset is available from https://github.com/xhzhang0377/MIV-Dataset (accessed on 19 April 2025).

## 2. Related Work

### 2.1. Deep-Learning-Based IVIF Method

Deep learning technology is commonly used in IVIF tasks and typically improves fusion quality by constructing more reasonable loss functions. For instance, Liu et al. [27] developed a coupled contrast constraint in the loss function that preserves the typical characteristics of IVIs. Zhang et al. created a general model [28] to perform various image fusion tasks by adjusting the weights of each intensity loss term. U2Fusion uses information measurement and feature extraction [29] to automatically assess the IVIs’ significance. To adjust to a range of image fusion tasks, MUFusion [30] utilizes the intermediate training outcomes as supplementary supervision signals. In recent years, the advancements in diffusion models [31,32] have brought new perspectives to image fusion. Zhao et al. [31] decomposed the fusion task into an unconditional generation component and a maximum likelihood estimation step. To enhance color fidelity, Yue et al. [32] utilized a diffusion-based model to generate the distribution of multi-channel input data. However, diffusion-based models require repeated iterative denoising processes, leading to a substantial computational burden and limiting their practical utility at the current stage. Static fusion methods [33,34] typically employ fixed fusion strategies, lacking adaptability. For different application scenarios or data distributions, manual adjustment of fusion parameters is required.

Additionally, some CNN-based IVIF methods focus on the impact of scene brightness on the quality of the fused images. For example, PIAFusion [35] incorporates illumination probability for developing an illumination perception loss function. Song et al. designed an illumination adjustment network [36] aimed at enhancing night-time visual image brightness. IAIFNet introduces a salient target-aware module alongside an adaptive differential fusion module to refine the IVIF process [37]. Meanwhile, MLFFusion [38] features a regional illumination preservation module specifically to boost fusion algorithm performance in low-light conditions. Although these methods yield impressive imaging results in dark environments, brightness adjustment inevitably leads to overexposure issues in daylight scenarios. The proposed network addresses this issue by employing gradient maximization and pixel value maximization losses to ensure excellent imaging in night-time environments. Simultaneously, the auxiliary loss generated by the decoupling network was designed to prevent brightness imbalance in daytime settings, thereby enhancing the overall performance of the image fusion process across diverse lighting conditions.

Typical encoder-based IVIF technologies [14,15,39] exploit the results of each layer in the encoding network to build feature maps. Although some nested connections [15,39] are used in IVIF to help mitigate the loss of detailed information, the degradation of pixel information in the original input images is inevitable. To further improve fusion quality, Zhao et al. [40] constructed two optimization models from the iterative formulations of two conventional optimization models. Tang et al. proposed the DIVFusion [41] scene illumination decomposition network to remove deteriorated visible illumination features. Additionally, for pixel-level IVIF, SGFusion [42] creates a saliency-guided deep learning framework. CDDFuse processes low-frequency global features in IVIs by using long-range attention [43]. In addition, ResCC [44] uses the spatial cross-attention model, while GTMFuse [45] employs a group attention transformer mechanism, both achieving superior fusion effects for IVIs. Nonetheless, these methods fail to take into account the guidance provided by advanced visual tasks on the fusion outcomes and might not be well suited for real-world applications.

In the domain of GAN-based IVIF, FusionGAN [20] eliminates the need for manually designing complex activity-level measurements and fusion rules. GAN-FM features a full-scale jump-connected generator alongside two distinct Markovian discriminators [21]. Based on this, UMF-CMGR introduces a generative registration paradigm [46] designed to remove artifacts in fused images. Meanwhile, GANMcC simultaneously estimates the distributions of both the IVIs domains, employing a multi-classification discrimination mechanism to achieve a more balanced fusion result [47]. However, these methods face issues of training instability and poor robustness in practical applications. Subsequently, some approaches incorporate high-level visual task guidance into GAN networks, for instance, TarDAL [48] and AT-GAN [26], but these GAN-based fusion models lack the ability to perceive the typical feature regions of IVIs.

### 2.2. Transformer-Based IVIF

With the self-attention mechanism, transformer can establish long-distance dependencies and effectively acquire global contextual information. Consequently, IVIF strategies have made use of transformer. For instance, SwinFusion [49] integrates a self-attention-based intra-domain fusion unit and a cross-attention-based inter-domain fusion unit to effectively merge the complementary features of IVIs. This design allows for efficient feature integration across different domains. DATFuse [50] introduces a dual-attention residual module to identify the salient characteristics of both visible and infrared inputs. AcFusion [51] employs ACmix, which combines multi-head self-attention and convolution to improve the model’s global modeling ability. Following this, leveraging the advantage of transformer in reducing parameter quantities, transformer has been increasingly applied in IVIF. Mustafa et al. applied a transformer block to extract high-frequency domain-specific information from source IVIs [52]. CDDFuse uses a light transformer module [43] that processes the low-frequency global features of IVIs. GTMFuse [45] integrates a group attention transformer module into its encoder, which combines window, channel, and fixed-direction stripe attention seamlessly for better IVIF. Furthermore, TGFuse [25] learns the global relationships among IVIs in complex scenes using a transformer fusion module. Additionally, Wu et al. proposed a multi-source image fusion method based on a fully connected transformer [53], which enhances the interaction and information transfer between different modalities through a fully connected mechanism. Although these methods can reduce the impact of redundant information to some extent, they have limitations in reducing the number of parameters, making it difficult to adapt to real-time fusion and vision tasks.

### 2.3. Advanced Task-Guided IVIF

Some IVIF methods are driven by advanced visual tasks, thereby compelling the fused images to encompass richer semantic information. For example, a salient object mask was introduced into the loss function to guide the optimization of STDFusionNet [16] to achieve the goal of salient object detection. Segmentation results are used in SuperFusion [54] and SeAFusion [23] to constrain semantic loss, thus guiding the high-level semantic information to flow back to the IVIF model. Moreover, detection-driven loss is created in DetFusion [24], which uses a significant target enhancement method [55] to optimize the IVIF task. However, these methods overlook the characteristics of the underlying original images during the generation of fused images. Additionally, a GAN-based method, TarDAL [48], uses bilevel optimization for detection-oriented fusion. Through the semantic transformation module and instance attention module, AT-GAN [26] suppresses redundant visible and infrared features. Nevertheless, the single-discriminator GAN fusion network always leads to insufficient preservation of infrared target information in the fusion results. Other methods [56,57] take into account the requirements of downstream tasks, such as object detection and classification. The former combines rolling guidance filtering with gradient saliency maps to improve fusion performance, while the latter employs a pseudo-supervised generative adversarial network to enhance detail preservation and image quality. Additionally, the mask-guided Mamba fusion method [58] uses detection results from another modality to generate a mask map, which is then used to cover the intermediate feature maps of the current modality, focusing more on the target regions during the fusion process. However, the multiple convolutional layers and selective scanning involved in deep state–space models may increase the computational burden.

## 3. Proposed Method

### 3.1. Network Construction

**Figure 1:**
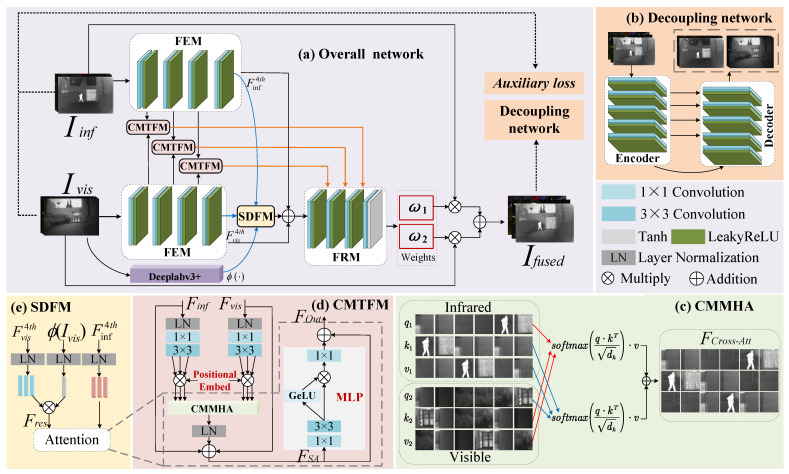
The architecture of the proposed network and related modules. (**a**) The network architecture of the proposed DSSFusion, (**b**) the framework of the decoupling network, (**c**) the computation of CMMHA, (**d**) the architecture of CMTFM, and (**e**) the architecture of SDFM.

The framework overview of the proposed DSSFusion is shown in Figure 1a. Firstly, the IVIs are separately input into a feature extraction module (FEM), to extract the shallow and deep features of the source images. The FEM contains four feature extraction layers. Each feature extraction layer comprises a 3 × 3 convolution and a LeakyReLU activation layer. Then, the first three layers of features from both modalities are correspondingly input into the CMTFM for shallow feature fusion. As shown in Figure 1d, the CMTFM utilizes the cross-attention mechanism of transformer to facilitate the interactive fusion between infrared and visible features. Subsequently, the infrared and visible features from the fourth feature extraction layer, together with the segmentation feature mask generated by the Deeplabv3+ network [59], are input into the SDFM (Figure 1e) to guide deep feature fusion with the segmentation network. Furthermore, the output features of SDFM, along with the fourth-layer infrared and visible features extracted in the FEM module, are concatenated and then fed into the feature reconstruction module (FRM), to supplement detailed information and reconstruct the fused features. Specifically, the input of the last three feature reconstruction layers includes the features from the previous reconstruction layer as well as the corresponding shallow fusion features from the CMTFM.

In the FRM, each of the first three feature reconstruction layers contains a 3 × 3 transposed convolutional layer and a LeakyReLU activation layer. The cross-modal fusion features after the first three layers of feature reconstruction are denoted as $F_{3\mathrm{th}}^{\mathrm{rec}}\in R^{H\times W\times 16}$. In the fourth layer of feature reconstruction, $F_{3\mathrm{th}}^{\mathrm{rec}}$ is first passed through a 3 × 3 convolutional layer to achieve channel compression, generating a two-channel tensor $\omega \in R^{H\times W\times 2}$. Then, the tensor values are constrained to the symmetric interval [−1, 1] through a Tanh activation function. A linear translation and scaling operation is introduced to adjust the dynamic range of the tensor to [0, 1], satisfying the probabilistic constraint for weight coefficients. Subsequently, ω is split along the channel dimension to obtain the infrared modality weight $\omega_{1}\in R^{H\times W\times 1}$ and the visible modality weight $\omega_{2}\in R^{H\times W\times 1}$. Finally, the two weights are multiplied point-wise with their corresponding original images and then added together to produce the fused image $I_{\mathrm{fused}}$. Dynamic adaptive weighting of cross-modal features helps achieve the optimal fusion of thermal radiation targets and visible image texture details.

In the training process, to further improve the quality of the fused image, $I_{\mathrm{fused}}$ is decomposed into new IVIs by the decoupling network (Figure 1b). The decoupled IVIs, along with the original images, are used to compute the auxiliary loss. Additionally, gradient loss and pixel loss are calculated between the fused image and the source images. These three types of losses are combined to jointly guide the training of DSSFusion.

### 3.2. Modules

#### 3.2.1. Cross-Modality Transformer Fusion Module

The architecture of the CMTFM is shown in Figure 1d. After applying LayerNorm to the features of the IVIs, the obtained feature maps undergo a 1 × 1 convolution and a 3 × 3 convolution. After the above operations, the channel number of the infrared and visible features is three times larger. Then, each modal feature is split into three parts along the channel dimension, which are used as the query, key, and value vectors. Taking the visible feature $F_{\mathrm{vis}}$ as an example, the operation proceeds as follows:

(1) $$q_{vis},k_{vis},v_{vis}=S\left({\mathrm{Conv}}_{3}^{3C,3C}\left({\mathrm{Conv}}_{1}^{C,3C}\left(LN\left(F_{vis}\right)\right)\right)\right)$$

where $q_{\mathrm{vis}}$, $k_{\mathrm{vis}}$, and $v_{\mathrm{vis}}$ are the query, key, and value of visible features, respectively. $F_{\mathrm{vis}}$ denotes the visible feature input to the CMTFM. LN(·) and S(·) denote the layer normalization and split operation along the channel dimension, respectively. ${\mathrm{Conv}}_{1}^{C,3C}\left(\cdot \right)$ signifies a 1×1 convolution with an input channel of *C* and an output channel of 3C. ${\mathrm{Conv}}_{3}^{3C,3C}\left(\cdot \right)$ implies a 3×3 convolution, with input and output channels both being 3C. Afterward, $q_{vis}$, $k_{vis}$, and $v_{vis}$ are embedded with learnable positional encoding, and the resulting vectors are denoted as $q_{2}$, $k_{2}$, and $v_{2}$, respectively. Note that the operation for the infrared feature $F_{inf}$ input to the CMTFM is the same as that for $F_{vis}$, and the resulting vectors are named $q_{1}$, $k_{1}$, and $v_{1}$ after introducing learnable positional encoding.

Leveraging its strong ability to represent long-distance dependencies, the transformer module is adept at capturing global content information from original images. This makes it particularly suitable for extracting nuanced features from IVIs. Consequently, the infrared and visible feature vectors are fed to the cross-modal multi-head attention (CMMHA, as shown in Figure 1c) to compute the cross-attention of the infrared and visible features, which can be formulated as follows:

(2) $$F_{Cross-Att}=\mathrm{Softmax}\left(\frac{q_{2}\cdot {k_{1}}^{T}}{\sqrt{d_{k}}}\right)\cdot v_{1}+\mathrm{Softmax}\left(\frac{q_{1}\cdot {k_{2}}^{T}}{\sqrt{d_{k}}}\right)\cdot v_{2}$$

where $F_{Cross-Att}$ denotes the cross-attention feature. $q_{1}$, $k_{1}$, and $v_{1}$ are the vectors from the infrared modality, and $q_{2}$, $k_{2}$, and $v_{2}$ are the vectors from the visible modality. $d_{k}$ represents the dimension of *k*.

Moreover, $F_{Cross-Att}$ is reshaped to map back to the input feature of the CMTFM, followed by layer normalization for decrease dimensions and two residual additions with $F_{inf}$ as well as $F_{vis}$ for complementing the detailed information of the IVIs. The above operation is formulated as

(3) $$F_{SA}=LN\left(F_{Cross-Att}\right)+F_{vis}+F_{inf}$$

where $F_{SA}$ denotes the feature map.

Cross-attention is effective in SwinFusion [49], so two separate MLPs are used for each modality without deeply mixing the two modalities. To reduce the number of parameters and enhance the recombination of different modal features, we adopt a shared MLP to further transform the fused features. The shared MLP introduces non-linear factors in the hidden layers, enabling the network to learn complex patterns and enhance the model’s generalization ability. Specifically, after undergoing a 1 × 1 convolution and a 3 × 3 convolution, $F_{SA}$ is split into two parts along the channel dimension, which can be formulated as

(4) $$F_{1},F_{2}=S\left({\mathrm{Conv}}_{3}^{2C/R,2C/R}\left({\mathrm{Conv}}_{1}^{C,2C/R}\left(F_{SA}\right)\right)\right)$$

where $F_{1}$ and $F_{2}$ denote the features after splitting. ${\mathrm{Conv}}_{1}^{C,2C/R}\left(\cdot \right)$ means a 1×1 convolution with an input channel of *C* and an output channel of 2C/R. ${\mathrm{Conv}}_{3}^{2C/R,2C/R}\left(\cdot \right)$ implies a 3×3 convolution with input and output channels both being 2C/R.

After that, feature $F_{1}$ is passed through a GeLU activation function and then multiplied element-wise with the other part, $F_{2}$. Then, the channel dimension is adjusted using a 1 × 1 convolution. Finally, an addition is constructed among MLP and $F_{SA}$, and the output of the CMTFM can be formulated as

(5) $$F_{CMTFM}^{\mathrm{out}}={\mathrm{Conv}}_{1}^{C/R,C}\left(\mathrm{GeLU}\left(F_{1}\right)⊙F_{2}\right)+F_{SA}$$

where $F_{CMTFM}^{\mathrm{out}}$ and ⊙ denote the output feature of the CMTFM, and element-wise multiplication, respectively. ${\mathrm{Conv}}_{1}^{C/R,C}\left(\cdot \right)$ means a 1×1 convolution with an input channel of C/R and an output channel of *C*. GeLU(·) means the GeLU activation function.

#### 3.2.2. Semantic-Driven Fusion Module

To assist the proposed method in understanding the boundaries and internal structures of different targets within images, a pre-trained semantic segmentation model Deeplabv3+ [59] is employed in the deep feature fusion process. The encoding structure of Deeplabv3+ consists of a ResNet-101 backbone and an atrous spatial pyramid pooling (ASPP) module. The ResNet-101 backbone extracts image features to generate high-level semantic features. The ASPP module captures multiscale semantic information using the high-level semantic feature maps generated by ResNet-101. In the tail of the encoder, the high-level semantic features of ASPP and the low-level features from ResNet-101 are combined along the channel dimension. Finally, in the decoding stage, the merged features undergo bilinear upsampling and mapping to produce the final segmentation feature map. The segmentation map is then used as a feature mask to further enhance the semantic information of the fused feature in SDFM (Figure 1e). As shown in Figure 1a, after the fourth feature extraction layer, the deep features of both modalities, along with the segmentation feature mask produced by Deeplabv3+, are input into the SDFM. The SDFM uses prior semantic knowledge to guide the fusion of deep features, which better preserves detailed information during the fusion. Specifically, we enhance the visible modality’s query with the segmentation features to ensure that semantic information is maximally preserved during the fusion process. This process can be formulated as

(6) $$q_{vis}^{4th},k_{vis}^{4th},v_{vis}^{4th}=S\left(LN\left(F_{vis}^{4th}\right)\right)$$

where $F_{vis}^{4th}$ is the visible feature extracted after the fourth feature extraction layer. $q_{vis}^{4th}$, $k_{vis}^{4th}$, and $v_{vis}^{4th}$ are the query, key, and value obtained from $F_{vis}^{4th}$ after undergoing layer normalization and split operation.

After the visible image undergoes semantic segmentation Deeplabv3+ and normalization, it is multiplied with query $q_{vis}^{4th}$ to enhance the feature representation of the target region. This operation is formulated as

(7) $$F_{res}=LN\left(\phi \left(I_{vis}\right)\right)⊙q_{vis}^{4th}$$

where $F_{\mathrm{res}}$ and $I_{\mathrm{vis}}$ denote the visible feature after embedding semantic segmentation and the source visible image, respectively. ϕ(·) represents the pre-trained semantic segmentation operation Deeplabv3+.

After that, special visible features $F_{\mathrm{res}}$ and the infrared feature $F_{inf}^{4th}$ extracted from the fourth feature extraction layer are fed into CMMHA and LN, followed by two residual connections, and then through an MLP. Finally, after another residual connection, the output of SDFM is obtained.

#### 3.2.3. Decoupling Network

To ensure that the fused images generated by the proposed network contain rich multimodal information, we follow a key principle: if the fused image can recover the original multimodal information, then the fusion network demonstrates high-quality multimodal feature fusion capability. Therefore, we adopt a decoupling network based on the U-Net architecture. The decoupling network is devoted to decomposing the fused high-quality images into modality-specific features that closely resemble the distribution of the original image information. As illustrated in Figure 1b, the decoupling network comprises an encoder, a decoder, and multilayer skip connections. This decoupling network is used to evaluate and guide the fusion performance of the proposed CMTFM and SDFM.

The input of the decoupling network is fused images. As illustrated in Figure 1b, the encoder consists of five feature extraction layers. Each feature extraction layer is composed of a 3 × 3 convolutional layer and a LeakyReLU activation layer, with a convolutional stride of 1 and padding of 1. These five layers of feature extraction effectively compress the input fused images and extract deep features. Additionally, the decoder of the decoupling network is made up of five feature reconstruction layers. Each feature reconstruction layer is composed of a transposed convolutional layer with a kernel size of 3 and a LeakyReLU activation layer. Through five layers of feature reconstruction, the original size and number of channels of the images are gradually restored. In each feature reconstruction step, the feature from the encoder is skip-connected to the feature reconstruction layer. Finally, these features are reconstructed into a new pair of IVIs.

By training the decoupling network and minimizing the loss function, the information distribution of the decoupled infrared and visible feature maps can be made to approximate that of the source images. Notably, during the training phase of DSSFusion, the decoupling network is used to decompose the images generated by DSSFusion. Moreover, the differences between the decoupled images and the original images are quantified through the auxiliary loss, which guides the training of DSSFusion to guarantee the fused images contain more multimodal information.

### 3.3. Loss Function

#### 3.3.1. Loss Function of the Decoupling Network

The input of the decoupling network is fused images, and the output is the reconstructed IVIs. Since the input fused images are obtained from the source IVIs, the loss of the decoupling network is defined as

(8) $$L_{D}=\mu_{1}(∥\nabla I_{vis}-\nabla \hat{I_{vis}}{∥}_{1}+∥\nabla I_{inf}-\nabla \hat{I_{inf}}{∥}_{1})+\mu_{2}(∥I_{vis}-\hat{I_{vis}}{∥}_{1}+∥I_{inf}-\hat{I_{inf}}{∥}_{1})$$

where $L_{D}$ and $I_{inf}$ are the loss of training of the decoupling network and source infrared image, respectively. $\hat{I_{vis}}$ and $\hat{I_{inf}}$ denote the reconstructed IVIs obtained after the decoupling network decomposing $I_{fused}$. The symbol ${∥\cdot ∥}_{1}$ represents the $l_{1}$ norm. ∇ denotes the Sobel gradient operator, which forces the reconstructed images to contain more texture information with high fidelity. $\mu_{1}$ and $\mu_{2}$ are the balance weights.

#### 3.3.2. Joint Loss of DSSFusion

To ensure that the fused images comprehensively reflect multimodal information and enhance the performance of the fusion strategies, we adopt a joint loss to train the DSSFusion network, which includes the gradient loss, pixel loss, and auxiliary loss as follows:

(9) $$L_{join}=\lambda_{1}\cdot L_{grad}+\lambda_{2}\cdot L_{pixel}+\lambda_{3}\cdot L_{aux}$$

where $L_{grad}$, $L_{pixel}$, and $L_{aux}$ denote the gradient loss, the pixel loss, and the auxiliary loss generated by the decoupling network, respectively. These three losses are combined to form the joint loss, denoted as $L_{join}$, to guide the training of the proposed network for retaining more details and information from the multimodal images. Additionally, $\lambda_{1}$, $\lambda_{2}$, and $\lambda_{3}$ are weighting coefficients used to adjust the weight of each loss term in the joint loss.

Gradient loss helps to preserve more edge and texture information in the fused images, which is particularly important for multimodal image fusion. The gradient loss is expressed as

(10) $$L_{grad}=∥\nabla I_{fused}-\nabla I_{vis}{∥}_{1}+{∥\nabla I_{fused}-\nabla I_{inf}∥}_{1}$$

where $I_{fused}$ is the fused image generated by DSSFusion.

Pixel loss directly compares the differences between the fused image and each source image at each pixel point, enabling the fused image to be close to the source images at the pixel level. The pixel loss is expressed as

(11) $$L_{pixel}=\frac{1}{N}\sum_{k=1}^{N}\left(|I_{fused}\left(i\right)-I_{vis}\left(i\right)|+|I_{fused}\left(i\right)-I_{inf}\left(i\right)|\right)$$

where $L_{pixel}$ and *N* are the pixel loss and the total number of pixels in the image, respectively. $I_{fused}\left(i\right)$, $I_{vis}\left(i\right)$, and $I_{inf}\left(i\right)$ denote the intensity value at pixel *i* in the fused image, source visible image, and source infrared image, respectively. The symbol |·| refers to absolute value calculation, which ensures the consideration of only positive magnitudes for preserving the fine details and texture patterns in the fused image.

Additionally, the fused images generated by DSSFusion are decomposed by the trained decoupling network. The differences between the decomposed images and the original images are quantified using the auxiliary loss, denoted as $L_{aux}$, which motivates the fused images to contain more multimodal information. The auxiliary loss is calculated as

(12) $$L_{aux}=∥I_{vis}^{D}-I_{vis}{∥}_{1}+{∥I_{inf}^{D}-I_{inf}∥}_{1}$$

where $L_{aux}$ is the auxiliary loss. $I_{vis}^{D}$ and $I_{inf}^{D}$ denote the decomposed IVIs produced by the decoupling network.

## 4. Experiments

### 4.1. Experimental Setup

**Figure 2:**
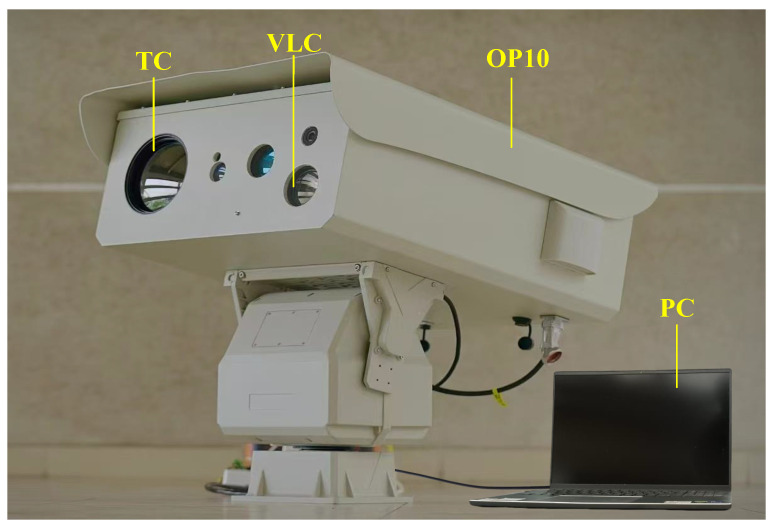
The visible and infrared imaging system.

As shown in Figure 2, we built an infrared and visible imaging system using Optical Pod No.10 (OP10) developed by our team to capture paired IVIs in maritime scenes. The data acquisition system comprised a visible light camera (VLC) with a resolution of 1920 × 1080, a thermal camera (TC) with a resolution of 1280 × 720 and a wavelength range of 8–14 µm, as well as a high-performance personal computer (PC). Both cameras interfaced with the PC through USB connections. The PC was responsible for executing tasks such as image registration, model inference, and result visualization. The MIV dataset [60] we created in this study (available online: https://github.com/xhzhang0377/MIV-Dataset (accessed on 19 April 2025) contains IVIs of maritime scenes during both daytime and night-time. Note that the infrared and visible image pairs in the proposed MIV dataset are aligned. During the alignment process, the common region of the infrared and visible images captured at the same time was first cropped, and then feature registration was performed using the modality conversion and registration method proposed in the UMF-CMGR [46]. Finally, we manually inspected and selected 109 pairs of aligned images to form the MIV dataset, which was also used to assess the performance of the proposed method.

### 4.2. Dataset Preparation

We performed a series of both qualitative and quantitative tests across four datasets to thoroughly assess the effectiveness of our proposed method. These datasets included three public datasets containing IVIs, namely, LLVIP [61], MSRS [62], and TNO [63], as well as the maritime dataset MIV. Specifically, the LLVIP dataset comprises aligned IVIs captured in night-time road scenes, being ideal for evaluating low-light vision techniques. The MSRS dataset images, taken under extremely low-light conditions, provided a rigorous performance test of the proposed method in challenging environments. Additionally, the TNO dataset offers multi-spectral night-time images from various military-related scenes with multiple targets, facilitating the assessment of the effectiveness of the proposed method in intricate scenarios.

Fusing infrared and visible modalities can enhance the safety and precision of maritime missions, such as port management, patrol enforcement, search and rescue, and accurate target detection and recognition. However, there are currently no aligned IVIs for maritime scenarios. As the dynamic changes in maritime environments and complex lighting conditions impose higher demands on fusion algorithms, we created the MIV dataset to evaluate the robustness and adaptability of the proposed method in practical applications.

### 4.3. Comparison Method and Evaluation Metrics

We compared the experimental results of DSSFusion with nine advanced fusion methods from recent years, including FusionGAN [20], GANMcC [47], RFN-Nest [39], UMF-CMGR [46], SuperFusion [54], MLFFusion [38], LRRNet [19], DATFuse [50], and ResCCFusion [44], to evaluate the fusion performance. The aforementioned image fusion methods and datasets are publicly available, and we used the parameters set in the original papers.

For quantitative evaluation, we employed seven metrics [64] to objectively assess the fusion performance of the comparison methods. They included standard deviation (SD), mutual information (MI), visual information fidelity (VIF), average gradient (AG), entropy (EN), edge preservation (Qabf), and space-frequency (SF). Specifically, SD provides a statistical measure of the distribution and contrast within a fused image. MI quantifies the shared information between source images. VIF evaluates the fidelity of information transfer from a human visual system perspective. AG measures the texture richness in a fused image. EN assesses the information content in a fused image using information theory principles. Qabf gauges the accuracy of edge information preservation. SF captures the spatial frequency characteristics present in a fused image. Higher values for these metrics generally indicate superior fusion performance. It is worth noting that the VIF values in this paper are the sum of the VIF between the fused image and the visible image and the VIF between the fused image and the infrared image. The numerical range of the evaluation metric VIF is between 0 and 2.

### 4.4. Training Details

**First-stage training:** In this stage, high-quality fused images were fed into the decoupling network, which output reconstructed IVIs. These reconstructed images were then compared with the original IVIs using the loss introduced in Section 3.3.1 to train the decoupling network. Thereby, the decoupling network decomposed the fused images and obtained modal features similar to the information distribution of the original images.

In detail, the nine fusion methods used in the experiments were applied to fuse the IVIs from the MSRS dataset. Then, 752 high-quality fused images were selected from these fused images as input for the decoupling network, including 376 daytime scene images and 376 night-time scene images. The selected fused images not only effectively retained the thermal imaging characteristics of the infrared images but also included rich details and color information from the visible images. For this training, the input images were divided into patches of size 64 × 64. Adam was used as the optimizer for the decoupling network, and the weight decay of the optimizer was set as $1\times 10^{-4}$. The learning rate of the optimizer was initially set as $1\times 10^{-3}$ and eventually decayed to $1\times 10^{-4}$. The batch size was set as 16, and the decoupling network was trained for $1\times 10^{3}$ epochs. The loss function parameters $\mu_{1}$ and $\mu_{2}$ were set as 50 and 20, respectively.

**Second-stage training:** We selected 752 pairs of IVIs from the MSRS dataset as input for the proposed DSSFusion, including 376 pairs of daytime scene images and 376 pairs of night-time scene images. The batch size was set as 8, and the input images were cropped into 64 × 64 patches for training. Additionally, all network parameters were updated using the Adam optimizer with a weight decay of $1\times 10^{-4}$, and the initial learning rate was set as $1\times 10^{-3}$. The loss function parameters $\lambda_{1}$, $\lambda_{2}$, and $\lambda_{3}$ were set as 50, 20, and 0.1, respectively.

During the first and second stages of training, the experiments were conducted using a NVIDIA GTX 2080 Ti GPU. The programming environment was Python 3.8 and PyTorch 2.1.1.

### 4.5. Experimental Results on Public Datasets

During the testing phase, the decoupling network was no longer used. Instead, DSSFusion served as an end-to-end network for inference, and the model parameters and weights obtained from the second training phase were directly utilized for testing. In this study, we selected some images for testing from three public datasets, specifically, 50 pairs from LLVIP at a resolution of 1280 × 1024 pixels, 56 pairs from MSRS at a resolution of 640 × 480 pixels, and 41 pairs from TNO at various resolutions. Note that the selected images were not used for training. The average quantitative results of the experiments on the three datasets are shown in Table 1, Table 2 and Table 3, respectively. By conducting tests on a diverse set of datasets, we comprehensively evaluated the performance of DSSFusion and verified its generalization capabilities across different scenarios.

#### 4.5.1. Fusion Results on LLVIP Dataset

Table 1 provides the quantitative results of seven metrics on the LLVIP test dataset. As shown in Table 1, the proposed DSSFusion demonstrates top two rankings across all seven metrics. Moreover, DSSFusion achieves the best VIF, AG, SF, and Qabf, which means that the fused images have satisfactory visual effects, as well as contain more texture and edge information. These improvements are attributed to the proposed SDFM and auxiliary loss. The large SD and EN values of MLFFusion indicate that its fused images have high contrast and a rich information content. Additionally, the large value of DATFuse for MI suggests that its fused images more faithfully reflect the information relationships with the original images.

**Figure 3:**
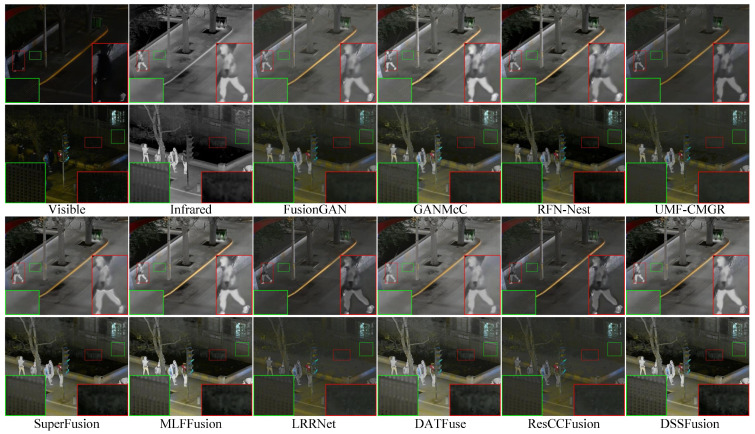
Qualitative comparison of DSSFusion with nine advanced methods on the LLVIP dataset.

To showcase the proposed method’s performance in low-light settings visually, we chose two sets of night-time images for subjective evaluation. The visualization outcomes are presented in Figure 3. For enhanced comparison, two regions of interest within each image are magnified for detailed examination. From Figure 3, it is evident that while all methods preserve prominent targets, certain approaches exhibit limitations in detail preservation. Specifically, DATFuse, MLFFusion, and SuperFusion do not clearly delineate the texture of the paving tiles. GANMcC, LRRNet, and RFN-Nest struggle to distinctly reveal the bushes obscured by darkness. Meanwhile, FusionGAN, UMF-CMGR, and ResCCFusion result in blurred representations of the bushes and fences. DSSFusion effectively integrates the complementary information from IVIs, simultaneously retaining a clear representation of both the bushes and the fence.

#### 4.5.2. Fusion Results on MSRS Dataset

**Figure 4:**
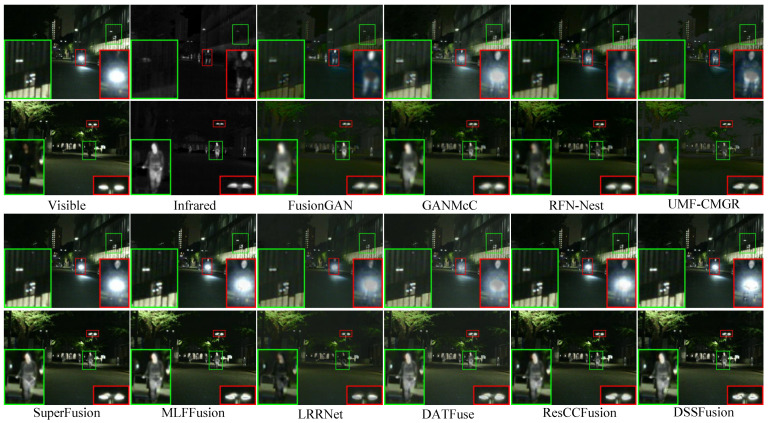
Qualitative comparison of DSSFusion with nine advanced methods on the MSRS dataset.

The quantitative results for the different methods on 56 pairs of images from the MSRS dataset are shown in Table 2. DSSFusion still demonstrates the best performance across seven metrics, ranking second only on the SD metric, proving that the proposed method is effective in extremely dark scenes. Additionally, MLFFusion maintains high contrast and competitiveness even in extremely dark environments. The qualitative comparison is shown in Figure 4. It can be noted that all methods maintain the intensity distribution of significant target areas. However, FusionGAN and UMF-CMGR lose information on the bicycle lamp in the fusion results and introduce artifacts, reducing the visual quality of the fused images. The images from GANMcC, LRRNet, and RFN-Nest have unclear human contours. In addition, DATFuse, SuperFusion, MLFFusion, and ResCCFusion fail to retain sharp edges. Nevertheless, DSSFusion fully incorporates the complementary and common features of the source images by integrating the CMTFM and SDFM. Consequently, DSSFusion effectively circumvents challenges such as thermal target degradation and texture blurring, which are conspicuously absent in its fused images. The validation through experiments on the MSRS dataset underscores DSSFusion’s proficiency in exceedingly low-light scenarios.

#### 4.5.3. Fusion Results on TNO Dataset

**Figure 5:**
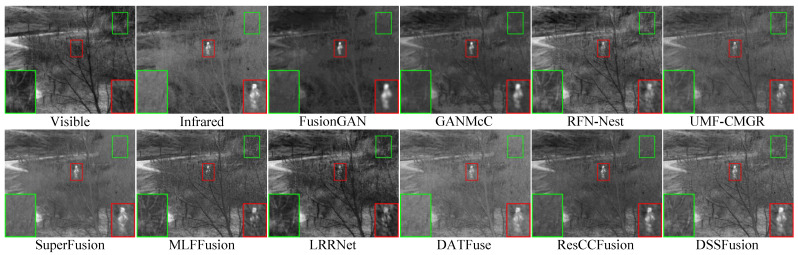
Qualitative comparison of DSSFusion with nine advanced methods on the field scene from the TNO dataset.

**Figure 6:**
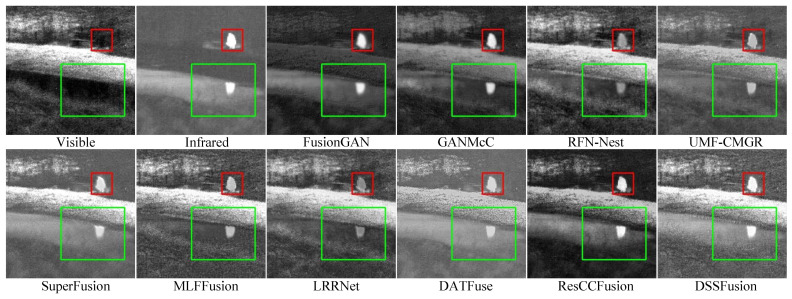
Qualitative comparison of DSSFusion with nine advanced methods on the bench scene from the TNO dataset.

The quantitative results of different methods on 41 pairs of images from the TNO dataset are shown in Table 3. The proposed DSSFusion performs quite well in multiple important aspects and can generate high-quality fused images. MLFFusion and LRRNet exhibit good contrast. ResCCFusion has advantages in terms of visual information fidelity and information content. The visual results are shown in Figure 5 and Figure 6. From the red boxes in Figure 5, FusionGAN and GANMcC blur the edges of thermal targets and exhibit severe spectral distortion in the background areas. LRRNet, MLFFusion, and RFN-Nest diminish the prominent targets. DATFuse, SuperFusion, and UMF-CMGR produce unclear trunk features. Only DSSFusion and ResCCFusion effectively retain the intensity of the prominent targets while preserving the texture details from the visible images. Comparable observations are evident in Figure 6. Note that, compared to other methods, DSSFusion effectively retains the texture details of visible images, as presented in the red and green boxes in Figure 5 and Figure 6.

### 4.6. Experimental Results on Actual Marine Datasets

**Experimental system.** The 109 pairs of images in the MIV dataset were used to validate the robustness of the proposed method in actual maritime scenarios. The average values measured across the seven evaluation metrics are shown in Table 4. SuperFusion has the greatest advantage in preserving the information relationship between the original images and fused images. MLFFusion exhibits excellent visual information fidelity. DSSFusion performs optimally in multiple key metrics, especially in terms of image contrast, detail preservation, and information content. The inference speed for image fusion on the MIV dataset is shown in Table 5, where DSSFusion has a faster inference speed than the nine methods.

**Figure 7:**
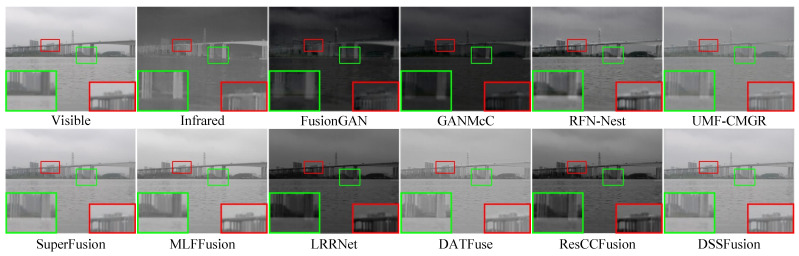
Qualitative comparison of DSSFusion with nine advanced methods on the daytime scene from the MIV dataset.

**Figure 8:**
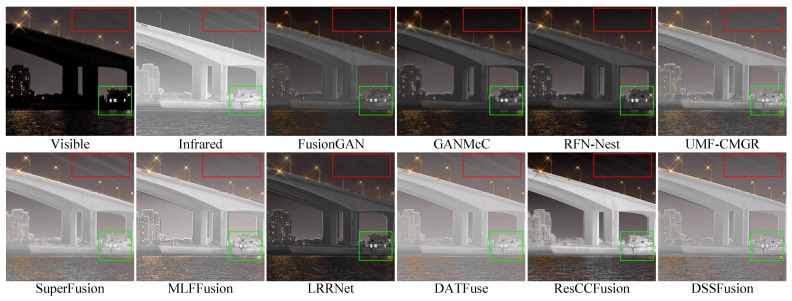
Qualitative comparison of DSSFusion with nine advanced methods on the night-time scene from the MIV dataset.

The fusion outcomes of the proposed method and compared methods are illustrated in Figure 7 and Figure 8. Notably, the MIV dataset was used exclusively for testing and did not participate in training. Figure 7 shows the fusion results in daytime maritime scenes. The images from FusionGAN, GANMcC, and LRRNet are generally darker and contain artifacts. RFN-Nest, UMF-CMGR, SuperFusion, and ResCCFusion overly integrate the intensity information from the infrared image while ignoring the detailed features of the visible image. MLFFusion and DATFuse fully integrate information from both modalities, but DSSFusion provides clearer details of the distant bridge and a more natural visual effect. Figure 8 shows the fusion results for night-time maritime scenes. FusionGAN, GANMcC, and RFN-Nest focus on the details of the visible image and fail to sufficiently fuse the infrared features, for example, losing the wire information in the top right corner of the infrared image. On the contrary, other methods (e.g., DATFuse, ResCCFusion, LRRNet, etc.) prioritize the intensity information in the infrared image, leading to incomplete feature integration of all targets in the image. The proposed DSSFusion generates higher-quality fused images, successfully integrating features such as the ships, buildings, and power lines in the image. Finally, the above experimental results indicate that DSSFusion has good target fusion performance under real-world conditions.

### 4.7. Ablation Study

To investigate the contribution and reliability of the proposed CMTFM, SDFM, weight fusion, and auxiliary loss, we conducted extensive ablation experiments on the TNO and MSRS datasets. The quantitative results are shown in Table 6. The best and suboptimal results are displayed in bold and underlined, respectively. In Exp. II, we eliminated weight fusion and directly obtained the fused image. In Exp. III, we replaced the CMTFM with an element-wise summation. In Exp. IV, we removed the auxiliary loss from Equation (9) by setting the hyperparameter of the last term to zero. In Exp. V, we removed the embedding DeepLabv3. The experiment where all the above elements were removed was recorded as Exp. I. On the contrary, the experiment with all modules added was the proposed DSSFusion (ours).

In Table 6, when the weight fusion is removed (Exp. II), most of the indicator values drop to suboptimal, indicating that some of the original information is partially compromised. Removing CMTFM leads to a decrease in the evaluation metrics (Exp. III), indicating that the absence of CMTFM results in insufficient fusion. In addition, it is validated that the auxiliary loss enables the fused image to contain more information from different modalities (Exp. IV), and the SDFM injects more semantic information into the network (Exp. V). Ours achieves the best and suboptimal performance across the seven evaluation metrics, indicating that DSSFusion combines the advantages of all designed modules.

### 4.8. Feature Visualization of Fusion Module

**Figure 9:**
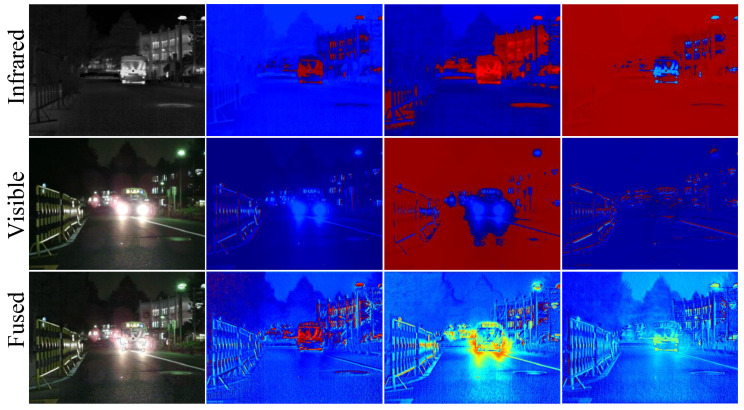
Visualization of feature maps learned from multi-modality image pairs in night-time scenarios. The initial column displays a sequential array of infrared, visible, and fused images. The subsequent three columns illustrate the feature maps associated with infrared, visible, and fused modalities across various channel dimensions.

To visually demonstrate the feature selection capability of the proposed SDFM under low-light conditions, we provide some thermal feature maps after the feature fusion stage in night-time scenes. These heatmaps are visualizations with a channel dimension of 128, shown in Figure 9. The first column of Figure 9 shows the original infrared image, the original visible image, and the fused image after SDFM. The next three columns display the feature heatmaps of these three modalities across different channel dimensions. From Figure 9, we can see that without using the SDFM, the differences between the infrared and visible features are evident. After adding the SDFM, the features of the infrared and visible modalities are fused and become clearer, indicating that the SDFM has a strong feature integration ability.

### 4.9. Visualization of the Decoupling Network

**Figure 10:**
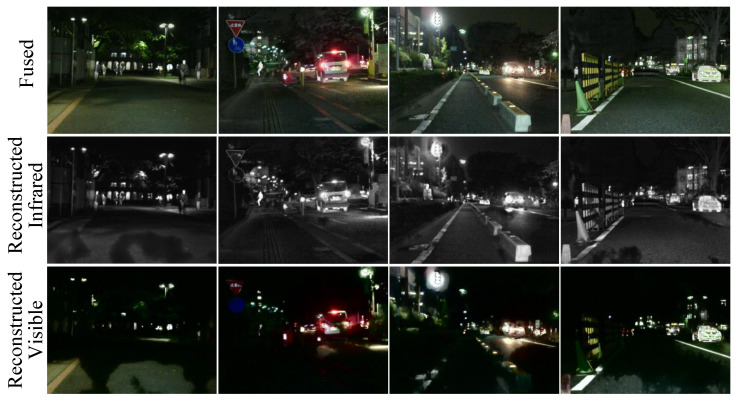
The fused image of night scenes from the MSRS dataset, along with visualizations of the reconstructed IVIs after decomposition by the decoupling network.

By visualizing the fused images, as well as reconstructed IVIs, we can better understand the working mechanism of the decoupling network and its ability to handle image details. As shown in Figure 10, the decoupling network is capable of effectively retaining and reconstructing important information from the input images.

## 5. Target Detection

On the Kaist dataset [65], we evaluate ddownstream tasks with the YOLOv8n [66] detector. We utilized the YOLOv8n model and fed the fusion results from different approaches straight into the detector for retraining to guarantee an equitable comparison. During the training phase of YOLO-v8n, the initial learning rate was set to 0.01, the batch size was configured to 16, the Adam optimizer was used, the number of epochs was set to 100, and the weight decay was set to 0.0005. The retrained models were then tested on a test set that was similarly randomly partitioned. Quantitative evaluations were obtained directly from the detector’s evaluation script, and all detector configurations remained true to their initial settings.

**Figure 11:**
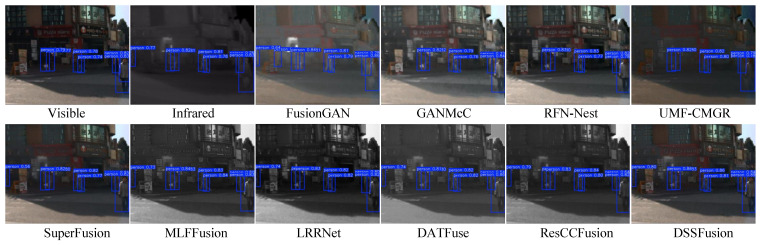
Qualitative comparison of target detection in daytime scenes on the Kaist dataset using different fused images.

**Figure 12:**
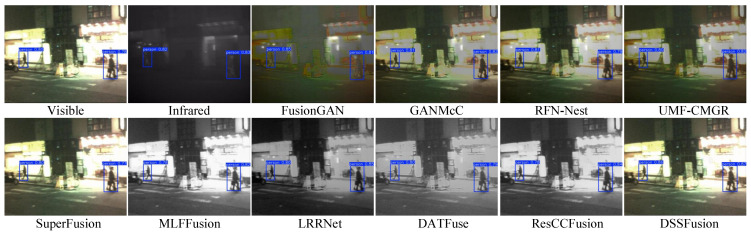
Qualitative comparison of target detection from night-time scenes from the Kaist dataset using different fused images.

We used four common evaluation metrics [67] for objective detection (namely, Precision, Recall, mAP@50, and mAP@[50:95]) to assess the image quality of ten fused images and original images. Precision denotes the accuracy of positive predictions, and Recall means the proportion of actual positives correctly identified. The mAP@50 is average precision at an Intersection over Union (IoU) threshold of 0.5, and the mAP@[50:95] is the average mAP across IoU thresholds from 0.5 to 0.95 in steps of 0.05. The quantitative results of the experiments are presented in Table 7. Higher scores on these metrics reflect superior image quality for detection tasks. Notably, DSSFusion achieves the highest accuracy in target detection, indicating its superior image quality. To further demonstrate this advantage, Figure 11 and Figure 12 provide visual examples of pedestrian detection accuracy across different images, showcasing detection with confidence scores exceeding 0.5.

Figure 11 shows the pedestrian detection results on daytime roads. By producing a high-contrast visual effect that is appropriate for the detection network, the fusion results from DSSFusion successfully highlight pedestrians, leading to improved detection performance. Conversely, methods like FusionGAN and MLFFusion have a tendency to blur pedestrian contours, which lowers detection confidence. Additionally, Figure 12 showcases night-time target detection, which better reflects DSSFusion’s ability to retain and utilize visible information. The pedestrian detection accuracy of the images generated by MLFFusion and ResCCFusion is lower in night scenes, which may be due to the impact of the lighting conditions on the model. In summary, the fused images produced by DSSFusion indicate optimal detection performance and the retention of rich visible information, meeting the requirements of the specific scenario.

## 6. Conclusions

We proposed and presented a decoupling and semantic segmentation-driven network for IVIF. To preserve informative features and avoid redundancy during the fusion process, we proposed CMTFM and SDFM, which learn rich hierarchical feature representations, better preserving and enhancing the key features of target objects. Additionally, a decoupling network was proposed to use auxiliary loss constraints to retain important features, achieving better visual effects. Unlike previous fusion methods, DSSFusion employs adaptive weight fusion to enable the automatic adjustment of fusion weights according to the data characteristics of different modalities. This successfully combines the structure and detail in visible images with the thermal properties of infrared photos. The results from both qualitative assessments and quantitative analyses demonstrated that DSSFusion markedly boosted the quality and utility of the fused images. Ablation experiments verified the effectiveness of the proposed method. Additionally, we extended the proposed network to maritime IVIF, which also achieved superior performance compared to other advanced methods. Furthermore, experiments on target detection demonstrated that the proposed method can be effectively applied to high-level visual tasks.

In future research, we will optimize the decomposition loss to further enhance the network’s ability to retain semantic features from multi-source images. We will also explore methods such as continual learning to improve the generalization of the proposed model across different fusion tasks.

## Figures and Tables

**Table 1:** Comparative analysis of DSSFusion and nine advanced methods on the LLVIP dataset. The best is indicated in bold and the second best with underlining.

Methods	SD	MI	VIF	AG	EN	Qabf	SF
FusionGAN	24.821	2.817	0.476	1.947	6.308	0.254	6.919
GANMcC	32.113	2.682	0.613	2.123	6.690	0.297	6.814
RFN-Nest	34.646	2.554	0.669	2.158	6.862	0.313	6.321
UMF-CMGR	29.379	2.682	0.521	2.504	6.462	0.347	9.915
SuperFusion	42.350	4.040	0.818	3.120	7.128	0.528	11.139
MLFFusion	**46.561**	3.739	0.960	4.085	**7.227**	0.675	13.953
LRRNet	24.867	2.348	0.564	2.467	6.145	0.424	9.108
DATFuse	39.850	**4.436**	0.839	3.036	7.082	0.514	12.331
ResCCFusion	41.006	3.920	0.849	2.841	7.093	0.519	10.851
DSSFusion	44.708	4.268	**0.985**	**4.134**	7.225	**0.697**	**14.384**

**Table 2:** Comparative analysis of DSSFusion and nine advanced methods on the MSRS dataset. The best is indicated in bold and the second best with underlining.

Methods	SD	MI	VIF	AG	EN	Qabf	SF
FusionGAN	17.145	1.843	0.413	1.209	5.250	0.138	3.875
GANMcC	24.349	2.376	0.682	1.835	5.832	0.338	5.227
RFN-Nest	21.588	2.141	0.574	1.266	5.389	0.242	4.337
UMF-CMGR	16.889	1.587	0.308	1.738	5.058	0.232	6.033
SuperFusion	32.159	3.173	0.917	2.570	5.903	0.589	8.504
MLFFusion	**33.189**	3.372	0.988	3.056	5.787	0.640	9.621
LRRNet	20.323	2.065	0.436	1.692	5.183	0.308	6.089
DATFuse	27.089	3.183	0.849	2.618	5.809	0.542	8.873
ResCCFusion	29.882	3.719	0.942	2.500	5.836	0.629	8.357
DSSFusion	32.714	**4.312**	**1.052**	**3.062**	**6.041**	**0.684**	**9.810**

**Table 3:** Comparative analysis of DSSFusion and nine advanced methods on the TNO dataset. The best is indicated in bold and the second best with underlining.

Methods	SD	MI	VIF	AG	EN	Qabf	SF
FusionGAN	30.781	2.333	0.424	2.418	6.557	0.234	6.272
GANMcC	33.423	2.280	0.532	2.520	6.734	0.279	6.111
RFN-Nest	36.940	2.129	0.561	2.654	6.966	0.333	5.846
UMF-CMGR	30.117	2.229	0.598	2.969	6.537	0.411	8.178
SuperFusion	37.082	3.417	0.686	3.566	6.763	0.476	9.235
MLFFusion	**41.412**	3.281	0.755	4.020	6.907	0.519	10.009
LRRNet	40.987	2.530	0.564	3.762	6.991	0.353	9.510
DATFuse	28.351	3.452	0.730	3.697	6.551	0.523	10.047
ResCCFusion	40.712	3.658	**0.828**	3.805	**7.000**	0.519	10.003
DSSFusion	39.215	**4.538**	0.809	**4.501**	6.994	**0.602**	**11.887**

**Table 4:** Comparative analysis of DSSFusion and nine advanced methods on the MIV dataset. The best is indicated in bold and the second best with underlining.

Methods	SD	MI	VIF	AG	EN	Qabf	SF
FusionGAN	30.005	3.242	0.403	2.248	6.550	0.252	8.288
GANMcC	30.345	3.273	0.459	1.911	6.425	0.246	6.364
RFN-Nest	27.932	3.095	0.536	2.057	6.510	0.319	6.576
UMF-CMGR	30.349	3.596	0.486	2.500	6.548	0.368	9.655
SuperFusion	31.060	**4.966**	0.508	2.816	6.571	0.504	10.233
MLFFusion	35.264	4.401	**0.672**	3.449	6.690	0.531	12.744
LRRNet	23.095	3.484	0.453	2.301	6.159	0.353	9.352
DATFuse	27.033	3.783	0.570	3.307	6.381	0.471	12.479
ResCCFusion	35.756	4.329	0.641	3.264	6.885	0.486	12.338
DSSFusion	**36.050**	4.871	0.658	**3.463**	**6.909**	**0.569**	**12.814**

**Table 5:** Comparison of running speeds for ten fusion methods on the MIV dataset. The lowest and second-lowest values are labeled in bold and underline, respectively.

Speed	Fusion GAN	GANMcC	RFN-Nest	UMF-CMGR	Super Fusion	MLF Fusion	LRRNet	DATFuse	ResCC Fusion	DSS Fusion
**run_time** **(second/each** **image pair)** **parms/(M)**	0.126	0.268	0.243	0.067	0.265	0.137	0.295	0.058	0.330	**0.015**
	1.326	1.860	2.730	0.629	0.196	1.181	0.049	**0.011**	0.817	0.801

**Table 6:** Average metric values of DSSFusion-component combinations on TNO and MSRS datasets. The activation status of individual components is represented as ✓ (included) and × (excluded). Bolded and underlined values indicate the best and second-best performance, respectively.

Modules	Components				TNO Dataset							MSRS Dataset						
	**Map**	**CMTFM**	**loss**	**SDFM**	**SD**	**MI**	**VIF**	**AG**	**EN**	**Qabf**	**SF**	**SD**	**MI**	**VIF**	**AG**	**EN**	**Qabf**	**SF**
I	×	×	×	×	36.509	3.014	0.626	3.760	6.804	0.472	8.841	31.460	2.654	0.776	2.669	5.538	0.527	8.672
II	×	✓	✓	✓	39.193	4.403	0.781	4.003	6.853	0.533	10.453	32.138	4.103	1.011	2.501	5.903	0.607	8.584
III	✓	×	✓	✓	37.724	2.703	0.684	3.894	6.827	0.499	9.577	31.032	2.704	0.796	2.612	5.651	0.537	8.648
IV	✓	✓	×	✓	36.046	3.028	0.627	3.740	6.771	0.473	8.891	30.812	2.548	0.762	2.642	5.435	0.529	8.769
V	✓	✓	✓	×	37.659	2.717	0.588	3.601	6.840	0.436	8.276	**35.151**	1.905	0.657	2.143	5.664	0.388	8.384
Ours	✓	✓	✓	✓	**39.215**	**4.538**	**0.809**	**4.501**	**6.924**	**0.602**	**11.887**	32.714	**4.312**	**1.052**	**3.062**	**6.041**	**0.684**	**9.810**

**Table 7:** The pedestrian detection efficiency of the fused images generated by various fusion methods on the YOLOv8n model. Bold and underline denote the best and second-best, respectively.

Methods	Precision	Recall	mAP@50	mAP@[50:95]
Visible	0.945	0.931	0.974	0.674
Infrared	0.946	0.944	0.978	0.689
FusionGAN	0.954	0.934	0.978	0.677
GANMcC	0.948	0.918	0.970	0.665
RFN-Nest	0.953	0.921	0.973	0.675
UMF-CMGR	0.945	0.930	0.974	0.672
SuperFusion	0.951	0.936	0.976	0.682
MLFFusion	0.948	0.934	0.973	0.681
LRRNet	0.955	0.928	0.977	0.679
DATFuse	0.952	0.937	0.977	0.683
ResCCFusion	0.955	0.923	0.975	0.679
DSSFusion	**0.961**	**0.949**	**0.983**	**0.709**

## Data Availability

The data underlying the results presented in this paper are not publicly available at this time but may be obtained from the authors upon reasonable request.

## References

[1] Jiang C., Ren H., Ye X., Zhu J., Zeng H., Nan Y., Sun M., Ren X., Huo H. (2022). Object detection from UAV thermal infrared images and videos using YOLO models. Int. J. Appl. Earth Obs. Geoinform..

[2] Ma W., Wang K., Li J., Yang S.X., Li J., Song L., Li Q. (2023). Infrared and visible image fusion technology and application: A review. Sensors.

[3] Zhang H., Xu H., Tian X., Jiang J., Ma J. (2021). Image fusion meets deep learning: A survey and perspective. Inf. Fusion.

[4] Li C., Zhu C., Huang Y., Tang J., Wang L. (2018). Cross-modal ranking with soft consistency and noisy labels for robust RGB-T tracking. Proc. Eur. Conf. Comput. Vis. (ECCV).

[5] Zhou W., Liu J., Lei J., Yu L., Hwang J.-N. (2021). GMNet: Graded-feature multilabel-learning network for RGB-thermal urban scene semantic segmentation. IEEE Trans. Image Process..

[6] Lu Y., Wu Y., Liu B., Zhang T., Li B., Chu Q., Yu N. Cross-modality person re-identification with shared specific feature transfer. Proceedings of the 2020 IEEE/CVF Conference on Computer Vision and Pattern Recognition (CVPR).

[7] Ma J., Zhou Y. (2020). Infrared and visible image fusion via gradientlet filter. Comput. Vis. Image Underst..

[8] Chen J., Li X., Luo L., Mei X., Ma J. (2020). Infrared and visible image fusion based on target-enhanced multiscale transform decomposition. Inform. Sci..

[9] Liu Y., Chen X., Ward R.K., Wang Z.J. (2016). Image fusion with convolutional sparse representation. IEEE Signal Process. Lett..

[10] Xing C., Wang Z., Ouyang Q., Dong C., Duan C. (2019). Image fusion method based on spatially masked convolutional sparse representation. Image Vis. Comput..

[11] Kong W., Lei Y., Zhao H. (2014). Adaptive fusion method of visible light and infrared images based on non-subsampled shearlet transform and fast non-negative matrix factorization. Infr. Phys. Technol..

[12] Bavirisetti D.P., Dhuli R. (2016). Two-scale image fusion of visible and infrared images using saliency detection. Infr. Phys. Technol..

[13] Ma J., Zhou Z., Wang B., Zong H. (2017). Infrared and visible image fusion based on visual saliency map and weighted least square optimization. Infr. Phys. Technol..

[14] Li H., Wu X. (2018). DenseFuse: A fusion approach to infrared and visible images. IEEE T. Image Process..

[15] Li H., Wu X., Durrani T. (2020). NestFuse: An infrared and visible image fusion architecture based on nest connection and spatial/channel attention models. IEEE T. Instrum. Meas..

[16] Ma J., Tang L., Xu M., Zhang H., Xiao G. (2021). STDFusionNet: An infrared and visible image fusion network based on salient target detection. IEEE Trans. Instrum. Meas..

[17] Ram Prabhakar K., Sai Srikar V., Venkatesh Babu R. DeepFuse: A deep unsupervised approach for exposure fusion with extreme exposure image pairs. Proceedings of the IEEE International Conference on Computer Vision.

[18] Qi J., Abera D.E., Fanose M.N., Wang L., Cheng J. (2024). A deep learning and image enhancement based pipeline for infrared and visible image fusion. Neurocomputing.

[19] Li H., Xu T., Wu X.-J., Lu J., Kittler J. (2023). LRRNet: A novel representation learning guided fusion network for infrared and visible images. IEEE T. Pattern Anal. Mach. Intell..

[20] Ma J., Yu W., Liang P., Li C., Jiang J. (2019). FusionGAN: A generative adversarial network for infrared and visible image fusion. Inform. Fusion.

[21] Zhang H., Yuan J., Tian X., Ma J. (2021). GAN-FM: Infrared and visible image fusion using GAN with full-scale skip connection and dual Markovian discriminators. IEEE Trans. Comput. Imaging.

[22] Li L., Shi Y., Lv M., Jia Z., Liu M., Zhao X., Zhang X., Ma H. (2024). Infrared and visible image fusion via sparse representation and guided filtering in laplacian pyramid domain. Remote Sens..

[23] Tang L., Yuan J., Ma J. (2022). Image fusion in the loop of high-level vision tasks: A semantic-aware real-time infrared and visible image fusion network. Inform. Fusion.

[24] Sun Y., Cao B., Zhu P., Hu Q. Detfusion: A detection-driven infrared and visible image fusion network. Proceedings of the 30th ACM International Conference on Multimedia.

[25] Rao D., Xu T., Wu X. (2023). TGFuse: An infrared and visible image fusion approach based on transformer and generative adversarial network. IEEE Transactions on Image Processing.

[26] Rao Y., Wu D., Han M., Wang T., Yang Y., Lei T., Zhou C., Bai H., Xing L. (2023). AT-GAN: A generative adversarial network with attention and transition for infrared and visible image fusion. Inform. Fusion.

[27] Liu J., Lin R., Wu G., Liu R., Luo Z., Fan X. (2024). Coconet: Coupled contrastive learning network with multi-level feature ensemble for multi-modality image fusion. Int. J. Comput. Vis..

[28] Zhang H., Ma J. (2021). SDNet: A versatile squeeze-and-decomposition network for real-time image fusion. Int. J. Comput. Vis..

[29] Xu H., Ma J., Jiang J., Guo X., Ling H. (2020). U2Fusion: A unified unsupervised image fusion network. IEEE T. Pattern Anal..

[30] Cheng C., Xu T., Wu X. (2023). MUFusion: A general unsupervised image fusion network based on memory unit. Inform. Fusion.

[31] Zhao Z., Bai H., Zhu Y., Zhang J., Xu S., Zhang Y., Zhang K., Meng D., Timofte R., Van Gool L. DDFM: Denoising diffusion model for multi-modality image fusion. Proceedings of the IEEE/CVF International Conference on Computer Vision.

[32] Yue J., Fang L., Xia S., Deng Y., Ma J. (2023). Dif-fusion: Toward high color fidelity in infrared and visible image fusion with diffusion models. IEEE T. Image Process..

[33] Heredia-Aguado E., Cabrera J.J., Jiménez L.M., Valiente D., Gil A. (2025). Static Early Fusion Techniques for Visible and Thermal Images to Enhance Convolutional Neural Network Detection: A Performance Analysis. Remote Sens..

[34] Vivone G., Deng L.-J., Deng S., Hong D., Jiang M., Li C., Li W., Shen H., Wu X., Xiao J.-L. (2025). Deep Learning in Remote Sensing Image Fusion: Methods, protocols, data, and future perspectives. IEEE Geosci. Remote Sens. Mag..

[35] Tang L., Yuan J., Zhang H., Jiang X., Ma J. (2022). PIAFusion: A progressive infrared and visible image fusion network based on illumination aware. Inform. Fusion.

[36] Song W., Gao M., Li Q., Guo X., Wang Z., Jeon G. (2024). Optimizing Nighttime Infrared and Visible Image Fusion for Long-haul Tactile Internet. IEEE Trans. Consum. Electron..

[37] Yang Q., Zhang Y., Zhao Z., Zhang J., Zhang S. (2024). IAIFNet: An Illumination-Aware Infrared and Visible Image Fusion Network. IEEE Signal Process. Lett..

[38] Wang C., Sun D., Gao Q., Wang L., Yan Z., Wang J., Wang E., Wang T. (2023). MLFFusion: Multi-level feature fusion network with region illumination retention for infrared and visible image fusion. Infrared Phys. Techn..

[39] Li H., Wu X., Kittler J. (2021). RFN-Nest: An end-to-end residual fusion network for infrared and visible images. Inform. Fusion.

[40] Zhao Z., Xu S., Zhang J., Liang C., Zhang C., Liu J. (2021). Efficient and model-based infrared and visible image fusion via algorithm unrolling. IEEE T. Circ. Syst. Vid..

[41] Tang L., Xiang X., Zhang H., Gong M., Ma J. (2023). DIVFusion: Darkness-free infrared and visible image fusion. Inform. Fusion.

[42] Liu J., Dian R., Li S., Liu H. (2023). SGFusion: A saliency guided deep-learning framework for pixel-level image fusion. Inform. Fusion.

[43] Zhao Z., Bai H., Zhang J., Zhang Y., Xu S., Lin Z., Timofte R., Van Gool L. CDDFuse: Correlation-driven dual-branch feature decomposition for multi-modality image fusion. Proceedings of the IEEE/CVF International Conference on Computer Vision.

[44] Xiong Z., Zhang X., Han H., Hu Q. (2024). ResCCFusion: Infrared and visible image fusion network based on ResCC module and spatial criss-cross attention models. Infrared Phys. Technol..

[45] Mei L., Hu X., Ye Z., Tang L., Wang Y., Li D., Liu Y., Hao X., Lei C., Xu C. (2024). GTMFuse: Group-attention transformer-driven multiscale dense feature-enhanced network for infrared and visible image fusion. Knowl. Based Syst..

[46] Wang D., Liu J., Fan X., Liu R. Unsupervised misaligned infrared and visible image fusion via cross-modality image generation and registration. Proceedings of the International Joint Conference on Artificial Intelligence.

[47] Ma J., Zhang H., Shao Z., Liang P., Xu H. (2020). GANMcC: A generative adversarial network with multiclassification constraints for infrared and visible image fusion. IEEE T. Instrum. Meas..

[48] Liu J., Fan X., Huang Z., Wu G., Liu R., Zhong W., Luo Z. Target-aware dual adversarial learning and a multi-scenario multi-modality benchmark to fuse infrared and visible for object detection. Proceedings of the IEEE/CVF Conference on Computer Vision and Pattern Recognition.

[49] Ma J., Tang L., Fan F., Huang J., Mei X., Ma Y. (2022). SwinFusion: Cross-domain long-range learning for general image fusion via swin transformer. IEEE/CAA J. Autom. Sin..

[50] Tang W., He F., Liu Y., Duan Y., Si T. (2023). DATFuse: Infrared and visible image fusion via dual attention transformer. IEEE T. Circuits Syst. Video Technol..

[51] Zhu H., Wu H., He D., Lan R., Liu Z., Pan X. (2023). AcFusion: Infrared and visible image fusion based on self-attention and convolution with enhanced information extraction. IEEE T. Cons. Electron..

[52] Mustafa H.T., Shamsolmoali P., Lee I.H. (2024). TGF: Multiscale transformer graph attention network for multi-sensor image fusion. Expert Syst. Appl..

[53] Wu X., Cao Z.-H., Huang T.-Z., Deng L.-J., Chanussot J., Vivone G. (2025). Fully-Connected Transformer for Multi-Source Image Fusion. IEEE T. Pattern Anal. Mach. Intell..

[54] Tang L., Deng Y., Ma Y., Huang J., Ma J. (2022). SuperFusion: A versatile image registration and fusion network with semantic awareness. IEEE/CAA J. Autom. Sin..

[55] Huo X., Deng Y., Shao K. (2022). Infrared and Visible Image Fusion with Significant Target Enhancement. Entropy.

[56] Li L., Lv M., Jia Z., Jin Q., Liu M., Chen L., Ma H. (2023). An effective infrared and visible image fusion approach via rolling guidance filtering and gradient saliency map. Remote Sens..

[57] Qi J., Abera D.E., Cheng J. (2024). PS-GAN: Pseudo Supervised Generative Adversarial Network With Single Scale Retinex Embedding for Infrared and Visible Image Fusion. IEEE J. Sel. Top. Appl. Earth Observ. Remote Sens..

[58] Wang S., Wang C., Shi C., Liu Y., Lu M. (2024). Mask-Guided Mamba Fusion for Drone-Based Visible-Infrared Vehicle Detection. IEEE Trans. Geosci. Remote Sens..

[59] Wang Y., Wang C., Wu H., Chen P. (2022). An improved Deeplabv3+ semantic segmentation algorithm with multiple loss constraints. PLoS ONE.

[60] MIV-Dataset. https://github.com/xhzhang0377/MIV-Dataset.

[61] Jia X., Zhu C., Li M., Tang W., Zhou W. LLVIP: A visible-infrared paired dataset for low-light vision. Proceedings of the 2021 IEEE/CVF International Conference on Computer Vision Workshops.

[62] MSRS Dataset. https://github.com/Linfeng-Tang/MSRS.

[63] TNO Dataset. https://figshare.com/articles/dataset/TNO_Image_Fusion_Dataset/1008029.

[64] Zhang X., Ye P., Xiao G. VIFB: A visible and infrared image fusion benchmark. Proceedings of the IEEE/CVF Conference on Computer Vision and Pattern Recognition Workshops.

[65] Hwang S., Park J., Kim N., Choi Y., So Kweon I. Multispectral pedestrian detection: Benchmark dataset and baseline. Proceedings of the IEEE Conference on Computer Vision and Pattern Recognition.

[66] Jocher G., Chaurasia A., Qiu J. Ultralytics YOLO. Software 2023, Version 8.0.0. https://github.com/ultralytics/ultralytics.

[67] Ren S., He K., Girshick R., Sun J. (2017). Faster R-CNN: Towards Real-Time Object Detection with Region Proposal Networks. IEEE Trans. Geosci. Remote Sens..

